# Supplementary material for: Topological synchronization of chaotic systems
Source: Sci Rep. 2022 Feb 15;12:2508. doi: 10.1038/s41598-022-06262-z (PMC8847423; doi:10.1038/s41598-022-06262-z)
Supplement: Supplementary file 1 — Supplementary Information 1. [file 41598_2022_6262_MOESM1_ESM.docx]

**Topological synchronization of chaotic systems**

**Supplementary material**

**Supplementary video 1**

video of the whole Topological synchronization and zipper effect process for the Logistic map system. General dimension D_q_ as function of the parameter q of master (blue) and slave (Red) attractors. As coupling k increases a zipper effect from the negative (q≤0) to the positive (q>0) part of D_q_ can be seen.

**Supplementary video 2**

Various details of the same synchronization process for the Logistic map system shown in supplementary video 1. The changes of the one-dimensional attractors as the coupling strength is being increased can be seen in the middle panel. Left side is the slave and right side is the master attractors. Upper panel, histogram of number of points in different areas of the attractors. Right lower panel, a segment of the x variable of the slave and the master oscillators vs time. Left lower panel, a plot of the x variable of the master vs the x variable of the slave.

**Supplementary video 3**

Topological synchronization and the zipper effect for high mismatched Rössler system. General dimension D_q_ as function of the parameter q of master (blue) and slave (red) attractors. As coupling k increases a zipper effect from the negative to the positive part of can be seen.

**Supplementary video 4**

Various details of the same synchronization process for high mismatched Rössler system shown in supplementary video 3. Where a 2D projection of the attractors can be seen in the upper panel as the coupling strength is being increased. Left side is the slave and right side is the master attractors. Middle left panel, a plot of the x variable of the master vs the x variable of the slave. Middle right panel, a plot of the y variable of the master vs the y variable of the slave. Right lower panel, a segment of z variables of both oscillators vs time. Left lower panel, a plot of the z variable of the master vs the z variable of the slave.

**Supplementary material note 1**

Both in logistic map system and in high mismatched Rössler system D_0_ is the first dimension that syncs in the q ≤ 0 region (see supplementary video 1 and supplementary video 2 at around k = 0.15 for Logistic map systems, and at around σ = 1.5 for Rössler systems in supplementary videos 3 and 4). Comparing the videos, one can see that when D_0_ is synced the structure of the slave attractor seems to become very similar to the master (compare between video 1 and 2 and also between video 3 and 4).
